# Association of a Health Equity Curriculum With Medical Students’ Knowledge of Social Determinants of Health and Confidence in Working With Underserved Populations

**DOI:** 10.1001/jamanetworkopen.2021.0297

**Published:** 2021-03-01

**Authors:** Nancy Denizard-Thompson, Deepak Palakshappa, Andrea Vallevand, Debanjali Kundu, Amber Brooks, Gia DiGiacobbe, Deborah Griffith, JaNae Joyner, Anna C. Snavely, David P. Miller

**Affiliations:** 1 Department of Internal Medicine, Wake Forest School of Medicine, Winston-Salem, North Carolina; 2 Department of Pediatrics, Wake Forest School of Medicine, Winston-Salem, North Carolina; 3 Division of Public Health Sciences, Wake Forest School of Medicine, Winston-Salem, North Carolina; 4 Medical Education, Wake Forest School of Medicine, Winston-Salem, North Carolina; 5 Department of Psychiatry, Wake Forest School of Medicine, Winston-Salem, North Carolina; 6 Department of Anesthesiology, Wake Forest School of Medicine, Winston-Salem, North Carolina; 7 Kaiser Permanente Bernard J. Tyson School of Medicine, Pasadena, California; 8 Forsyth Tech Community College, Winston-Salem, North Carolina

## Abstract

**Importance:**

National organizations recommend that medical schools train students in the social determinants of health.

**Objective:**

To develop and evaluate a longitudinal health equity curriculum that was integrated into third-year clinical clerkships and provided experiential learning in partnership with community organizations.

**Design, Setting, and Participants:**

This longitudinal cohort study was conducted from June 2017 to October 2020 to evaluate the association of the curriculum with medical students’ self-reported knowledge of social determinants of health and confidence working with underserved populations. Students from 1 large medical school in the southeastern US were included. Students in the class of 2019 and class of 2020 were surveyed at baseline (before the start of their third year), end of the third year, and graduation. The class of 2018 (No curriculum) was surveyed at graduation to serve as a control. Data analysis was conducted from June to September 2020.

**Exposures:**

The curriculum began with a health equity simulation followed by a series of modules. The class of 2019 participated in the simulation and piloted the initial 3 modules (pilot), and the class of 2020 participated in the simulation and the full 9 modules (full).

**Main Outcomes and Measures:**

A linear mixed-effects model was used to evaluate the change in the self-reported knowledge and confidence scores over time (potential scores ranged from 0 to 32, with higher scores indicating higher self-reported knowledge and confidence working with underserved populations). In secondary analyses, a Kruskal-Wallis test was conducted to compare graduation scores between the no, pilot, and full curriculum classes.

**Results:**

A total of 314 students (160 women [51.0%], 205 [65.3%] non-Hispanic White participants) completed at least 1 survey, including 125 students in the pilot, 121 in the full, and 68 in the no curriculum classes. One hundred forty-one students (44.9%) were interested in primary care. Total self-reported knowledge and confidence scores increased between baseline and end of clerkship (15.4 vs 23.7, *P* = .001) and baseline and graduation (15.4 vs 23.7, *P* = .001) for the pilot and full curriculum classes. Total scores at graduation were higher for the pilot curriculum (median, 24.0; interquartile range [IQR], 21.0-27.0; *P* = .001) and full curriculum classes (median, 23.0; IQR, 20.0-26.0; *P* = .01) compared with the no curriculum class (median, 20.5; IQR, 16.25-24.0).

**Conclusions and Relevance:**

In this cohort study of medical students, a dedicated health equity curriculum was associated with a significant improvement in students’ self-reported knowledge of social determinants of health and confidence working with underserved populations.

## Key Points

**Question:**

Is a longitudinal health equity curriculum associated with improved self-reported knowledge of the social determinants of health and confidence with working with underserved populations among US medical students?

**Findings:**

In this cohort study of 314 students, self-reported knowledge and confidence scores significantly increased over time for participants in both the pilot and full curriculum classes. Compared with students not exposed to the curriculum, those in the pilot and the full curriculum classes had significantly higher scores at graduation.

**Meaning:**

A longitudinal health equity curriculum that was integrated within clinical clerkships and partnered with community-based organizations was associated with an improvement in students’ self-reported understanding of the social determinants of health.

## Teaser

This cohort study assesses whether a health equity curriculum is associated with knowledge of social determinants of health and confidence with working with underserved populations among US medical students.

## Introduction

The social determinants of health (SDH)—the conditions in which people are born, work, live, and age—have a profound effect on morbidity and mortality.^1^ National organizations have emphasized that medical schools should teach trainees about the SDH and their effect on health disparities.^2,3,4,5^ Disruptions related to the coronavirus disease 2019 (COVID-19) pandemic and social unrest in the US have brought additional stressors and amplified underlying inequalities in educational and health systems. Thus, it is imperative that medical schools increase commitment and investment in teaching students about SDH and health equity, and medical school education can have an influence in reducing health disparities.^6,7^ Prior studies have found that students who attend medical schools that include health equity curricula are more likely to practice in underserved communities.^8,9,10^

An increasing number of US medical schools have begun to recognize the need for health equity curricula that include issues such as access to care, housing instability, and racial/ethnic bias.^11^ National organizations recommend that effective medical school health equity curricula should integrate public health with clinical care, engage with the community, and partner with key community organizations addressing patient needs.^4^ Also, these curricula should involve long-term evaluation to determine how they modify students’ behaviors and meet students’ needs.^12,13^

Although many schools have begun to implement health equity curricula, they are often limited to small classroom-based experiences without significant community involvement.^12,13,14^ Many are delivered as electives that reach only students with self-identified interest in health disparities,^15,16^ and only a few have reported student evaluation data.^12,13^ Effective examples of experiential health equity curricula that are woven into the medical school’s core curricula are needed.^12,14^

To address these gaps, we developed and implemented a longitudinal health equity curriculum for all students that was integrated into the third-year clinical clerkships. The curriculum combined didactic training with experiential learning by partnering with community organizations located throughout the city. We implemented the curriculum, and over a period of 3 years (2018-2020), assessed medical students’ self-reported competence and confidence working with underserved populations.

## Methods

### Study Design and Population

We developed and implemented a longitudinal health equity curriculum for third-year medical students at Wake Forest School of Medicine. Concurrent with the implementation, we conducted a longitudinal cohort study to evaluate the association of the curriculum with the students’ self-reported knowledge of the SDH and confidence working with underserved populations. The curriculum was integrated into the third year, when students begin clinical clerkship rotations (eg, internal medicine, surgery). All students in the classes of 2019 and 2020 participated in the curriculum and were invited to participate in the study. Students in the class of 2019 participated in the curriculum from June 2017 to June 2018. Students in the class of 2020 participated in the curriculum from March 2018 to March 2019.

The Wake Forest School of Medicine institutional review board approved the study with a waiver of signed informed consent. All students were informed in writing that their participation was voluntary and that by completing a study survey, they were agreeing to have their data used for study purposes. This study followed the Strengthening the Reporting of Observational Studies in Epidemiology (STROBE) reporting guideline for cohort studies.

### Health Equity Curriculum

**Figure 1.**
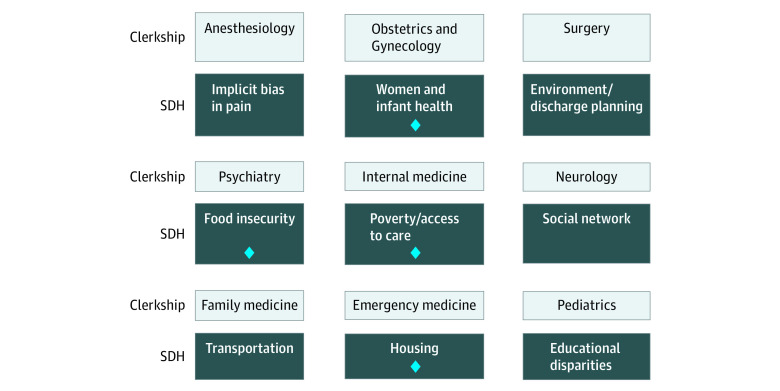
Integration of Social Determinants of Health (SDH) Modules Within the Third-Year Clerkships Diamonds indicate which modules include a specific community partner to provide experiential learning opportunity.

The curriculum was based on the 3 domains (education, community, and organization) set forth by the National Academies of Sciences, Engineering, and Medicine’s Framework for Educating Health Professionals to Address the Social Determinants of Health.^4^ The curriculum leadership met with all of the clinical clerkship directors individually to identify which SDH may be most relevant to students’ experiences during the rotation. The curriculum course directors then met with community organizations throughout the city to identify organizations that would be interested in participating and had the capacity to include students. The clerkship directors, leaders from the community organizations, and the curriculum directors then developed an experiential learning activity together that would be mutually beneficial for the students and community organizations. The full curriculum consisted of a health equity simulation and a series of 9 modules that exposed students to different SDH (Figure 1).

During their third-year orientation week, all students participated in the simulation over a 2-day period. The health equity simulation is an experiential learning activity in which learners take on the role of a community member. Learners experience 3 weeks (represented in three 15-minute blocks) in the person’s life through written assignments, learning tasks, and reflection on the experiences of their character. For example, 1 character is a man experiencing homelessness who has to find a way to attend a doctor’s appointment and obtain his necessary medications. Following the simulation, students come together as a large group to discuss what they learned.

The format of each of the modules is a prelearning activity (eg, an online or in-person lecture), an experiential activity, and an evaluation assignment (eg, reflection piece or short presentation) (eFigure in the Supplement). To provide students robust, contextualized learning experiences, we paired 1 SDH with each of the required clerkships through collaboration with the clerkship directors. We partnered with community organizations throughout the city to create experiential learning activities for students (eg, delivering meals in low-income neighborhoods). During the planning, the curriculum directors and the community organizations identified specific goals and activities for the students, and the curriculum directors frequently attended the activities to ensure all students received a similar experience. The class of 2019 participated in the simulation and piloted the initial 3 modules (pilot curriculum class). The initial 3 modules included internal medicine and poverty/access to care, psychiatry and food insecurity, and pediatrics and educational disparities. The class of 2020 participated in the simulation and the full 9 modules (full curriculum class).

### Survey and Data Collection

Through a detailed review of the literature and consultation with outside experts,^17,18,19,20,21,22,23,24,25,26^ we developed a survey to measure students’ self-reported knowledge of the SDH and confidence working with underserved populations. The survey consisted of 8 questions (eTable in the Supplement) measured on a 5-point Likert scale (0-4). We summed the 8 items to create a total score ranging from 0 to 32, with higher scores indicating higher knowledge of the SDH and confidence working with underserved populations. We found high internal consistency among the 8 questions and across domains with a Cronbach α > 0.8.

Students who participated in the curriculum (pilot and full) were asked to complete the survey at 3 separate points over a 2-year period (baseline, end of the third year, and graduation) (Table 1). Students completed baseline surveys before the start of the curriculum. We chose these points to evaluate changes from baseline to the end of the curriculum (at the end of the third year) and to determine whether these self-reported changes were sustained through graduation. Given that students are likely to have an increase in their knowledge and confidence by participating in their clinical rotations even without a dedicated curriculum, we also surveyed students in the class of 2018 (No curriculum class) at the time of their graduation to serve as a control. The class of 2018 did not receive any of the curriculum. All surveys were completed in person on paper-based forms except for the graduation survey for the full curriculum class, which was conducted online using REDCap, an online web application for managing and building surveys, because of the COVID-19 pandemic limiting in-person gatherings. All survey data were collected anonymously, with each student receiving a unique study identifier. The curriculum directors were not present at the time of data collection and were blinded from individual survey responses.

**Table 1.** Curriculum and Survey Timeline

Curriculum	Baseline survey	Year 3 curriculum	End of third-year survey	Graduation
No	NA	None	NA	June 2018
Pilot	June 2017	Health equity simulation and 3 modules	June 2018	June 2019
Full	March 2018	Health equity simulation and 9 modules	March 2019	June 2020

Abbreviation: NA, not applicable.

Students also completed a demographic survey including self-reported sex (male, female, or not reported), race/ethnicity (non-Hispanic White, Hispanic White, Black/African American, Asian/Pacific Islander, and other/mixed), and prior experience working with underserved populations (ie, with family, school, work, or volunteering) on a 5-point Likert scale (0, none at all and 4, a lot) at baseline. During the end of the third-year survey, we asked students their career interest (primary, specialty, or undecided), considering that they would be more likely to know their career plans at the end of the third year than the beginning. All demographic data for the class of 2018 were collected at graduation, because that was the only time the class completed a survey.

### Statistical Analysis

Data were analyzed from June to September 2020. We used χ^2^ tests to evaluate for differences in covariates between the 3 cohorts (no, pilot, and full). Survey ratings before the third year, at the end of the third year, and at graduation are reported using frequency statistics, and we used analysis of variance to test for differences in survey scores in unadjusted analyses. For our main analysis, we used a linear mixed-effects model and evaluated the interaction between time and cohort to determine the change in the total self-reported knowledge/confidence score over time within cohorts, controlling for sex, race/ethnicity, and prior experience, with individual students as the random intercept. We used estimated marginal means of the interaction between time and cohort to evaluate for differences in each individual cohort over time. Demographic data for sex, race/ethnicity, career interest, and prior experience were reported using descriptive statistics. Because of the small numbers of individual participants in each category, we dichotomized prior experience (0-2, none/little; 3-4, a lot) for the multivariable model. In secondary analyses, an independent-samples Kruskal-Wallis test was conducted to compare scores at graduation between the no, pilot, and full curriculum cohorts. a post hoc analysis was conducted to identify between-group differences in total scores. All analyses were completed using SPSS software, version 26 (IBM Corp). We used 2-sided hypothesis tests, and we considered α < .05 to be statistically significant.

## Results

### Study Population Characteristics

A total of 314 students were included in this study (160 women [51.0%], 205 [65.3%] non-Hispanic White participants). Of the 246 students who received any part of the health equity curriculum, 125 from the pilot curriculum and 121 from the full curriculum completed at least 1 survey. In the pilot curriculum class, 118 of 125 (94.4%) completed the baseline survey, 117 of 125 (93.6%) completed the end-of-third-year survey, and 77 of 121 (63.6%) completed the graduation survey. In the full curriculum class, 116 of 121 (95.9%) completed the baseline survey, 93 of 121 (76.9%) completed the end-of-third-year survey, and 104 of 114 (91.2%) completed the graduation survey. A convenience sample of 68 of 107 students (63.6%) from the No curriculum class completed the survey at graduation. Overall, 141 respondents (44.9%) reported being interested in pursuing primary care (41 [60.3%] in the no curriculum group, 49 [39.2%] in the pilot group, and 51 [42.1%] in the full curriculum group) (Table 2). There were no significant differences in sex, race/ethnicity, prior experience, or career interest between the cohorts.

**Table 2.** Demographic Characteristics for the 3 Cohorts

Characteristic	Students, No. (%)		
	**No curriculum (n = 68)**	**Pilot curriculum (n = 125)**	**Full curriculum (n = 121)**
Sex			
Male	37 (54.4)	64 (51.2)	53 (43.8)
Female	31 (45.6)	61 (48.8)	68 (56.2)
Race/ethnicity			
Non-Hispanic White	50 (73.5)	82 (65.6)	73 (60.3)
Hispanic	2 (2.9)	5 (4.0)	6 (5.0)
Black or African American	6 (8.8)	12 (9.6)	13 (10.7)
Asian or Pacific Islander	8 (11.8)	19 (15.2)	19 (15.7)
Other or mixed	2 (2.9)	5 (4.0)	9 (7.4)
Missing	0	0	1 (0.8)
Career interest^a^			
Primary	41 (60.3)	49 (39.2)	51 (42.1)
Specialty	27 (39.7)	41 (32.8)	38 (31.4)
Undecided	0	35 (28.0)	31 (25.6)
Missing	0	0	1 (0.8)
Prior experience^b^			
0	0	0	0
1	6 (8.8)	12 (10.2)	8 (6.6)
2	12 (17.6)	26 (22.0)	26 (21.5)
3	35 (51.5)	38 (32.2)	30 (24.8)
4	15 (22.1)	42 (35.6)	23 (19.0)
Missing	0	7 (5.6)	34 (28.1)

^a^ Career interest at the end of the clerkship year for 2019 (pilot) and 2020 (full) cohorts and at graduation for the 2018 (no curriculum) cohort.

^b^ Indicates prior experience working with underserved populations. Responses are scored on a 5-point Likert scale, with 0 indicating no experience and 4 indicating a lot.

### Change in Self-reported Knowledge and Confidence Over Time

In unadjusted analyses, self-reported knowledge and confidence scores for the pilot and full curriculum classes increased over time. Seventy-three of 121 students (60.3%) from the pilot curriculum completed the survey at all phases of the study. The mean total scale score significantly increased from baseline (15.4; 95% CI, 14.5-16.4) to end of clerkship (24.6; 95% CI, 23.6-25.5) with a difference of 9.2 (95% CI, 8.2-10.2; *P* < .001), and the mean total score increased from baseline to graduation (24.2; 95% CI, 23.1-25.3) with a difference of 8.7 (95% CI, 7.6-9.9; *P* < .001). The score did not significantly change from end of clerkship to end of school. Similarly, the full curriculum class (with 85 of 114 [74.6%] completing all 3 surveys) had a significant increase from baseline (15.1; 95% CI, 14.2-16.1) to end of clerkship (22.5; 95% CI, 21.4-23.5) with a difference of 7.4 (95% CI, 6.3-8.4; *P* < .001). The mean total score increased from baseline to graduation (23.0; 95% CI, 22.0-24.0) with a difference of 7.9 (95% CI, 6.9-8.9; *P* < .001) but did not change between end of clerkship and graduation.

In multivariable modeling, we found similar increases in self-reported knowledge and confidence scores over time (Table 3). We found the mean total sum score increased from baseline to end of clerkship (15.4 [95% CI, 14.5-16.3] vs 23.7 [95% CI, 23.0-24.4]; *P* = .001) and baseline to graduation (15.4 [95% CI, 14.5-16.3] vs 23.7 [95% CI, 22.9-24.5]; *P* = .001). There was no significant difference between the end of clerkship and graduation. We did find a small but statistically significant difference in the total scores between the 2 curricula, with the pilot curriculum mean score being higher than the mean score from the full curriculum (21.5 [95% CI, 20.6-22.3] vs 20.4 [95% CI, 19.6-21.2]; *P* = .04).

**Table 3.** Linear Mixed-Effects Model Evaluating Change in Total Confidence and Knowledge Score Over Time^a^

Variable	Estimated mean (95% CI)	*P* value
Time		
Baseline	15.4 (14.5-16.3)	.001
End of clerkship	23.7 (23.0-24.4)	
Graduation	23.7 (22.9-24.5)	
Curriculum		
Pilot	21.5 (20.6-22.3)	.04
Full	20.4 (19.6-21.2)	
Sex		
Male	20.4 (19.6-21.3)	.04
Female	21.4 (20.6-22.3)	
Race/ethnicity		
Non-Hispanic White	20.5 (19.8-21.1)	.32
Hispanic	20.7 (18.6-22.8)	
Black or African American	22.2 (20.7-23.7)	
Asian or Pacific Islander	20.8 (19.6-22.1)	
Other or mixed	20.5 (19.0-22.0)	
Prior experience with underserved populations		
Low	18.0 (17.4-18.7)	.001
High	21.6 (21.1-22.1)	
Estimated marginal means		
Cohort × time (Pilot)		
Baseline	15.4 (14.2-16.7)	.01
End of clerkship	24.7 (23.8-25.6)	
Graduation	24.3 (23.2-25.3)	
Cohort × time (Full)		
Baseline	15.4 (14.2-16.6)	.01
End of clerkship	22.7 (21.7-23.6)	
Graduation	23.2 (22.2-24.1)	

^a^ Linear mixed-effects model estimated means, 95% CIs, and *P* value for the fixed and interaction effects. The 8-item knowledge/confidence survey had a possible range of 0 to 32, with higher scores indicating greater knowledge or confidence.

### Difference Between Receiving Curriculum vs Not

**Figure 2.**
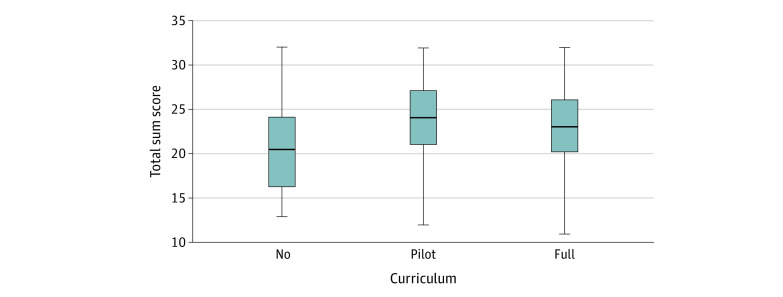
Difference in Graduation Survey Total Sum Scores Independent-samples Kruskal-Wallis analysis showing a significant difference in median end-of-year total sum score between students who had no curriculum, pilot curriculum, and full curriculum. The center lines indicate medians, and the upper and lower ends of the boxes indicate the 75th and 25th percentiles, respectively. Error bars indicate minimum and maximum.

In secondary analysis, we evaluated the difference in the total score at graduation between the classes who received the curriculum (pilot and full) compared with the class that did not receive the curriculum (no). We found statistically significant differences between the No curriculum (median, 20.5; interquartile range [IQR], third – first quartile, 16.25-24.0) and pilot curriculum (median, 24.0; IQR, 21.0-27.0; *P* = .001) and no curriculum and full curriculum (median, 23.0; IQR, 20.0-26.0; *P* = .01) classes (Figure 2). No statistically significant differences were noted in the median scores between the pilot and full curriculum classes at graduation.

## Discussion

In this study, we found that a dedicated longitudinal health equity curriculum was associated with a significant improvement in students’ self-reported knowledge of the SDH and confidence working with underserved populations. This increase in knowledge and confidence was sustained 1 year later. Additionally, students who received the curriculum reported higher total knowledge and confidence scores than students who did not receive the curriculum.

There has been growing interest among national health care organizations for health systems to address patients’ unmet social needs to reduce health disparities.^27,28,29,30,31^ Recognizing the need to prepare health care professionals to understand and mitigate the social and economic factors that lead to health disparities, medical schools are increasingly interested in implementing curricula to train students about the SDH and health disparities.^11,12,13^ Despite the growing interest, few curricula are currently available, and the curricula that have been developed are often limited in duration (<6 weeks) or only offered to a select number of students.^12,13^ Our school’s leadership was committed to ensuring all students had an understanding about the social and economic factors that lead to health disparities, so all students were required to complete the curriculum. Additionally, we integrated the curriculum within the third-year clerkships, when students would routinely begin to see patients, to provide further context in how the SDH affect patients’ health. Similar to other curricula,^16,18,32,33,34^ we found that the curriculum was associated with an improvement in students’ self-reported knowledge and confidence working with underserved populations. Additional studies, with longer follow-up extending through residency, are needed to determine the impact on future care.

The National Academies of Sciences, Engineering, and Medicine’s framework for educating health care professionals recommends working with the community to provide students training in the SDH.^4^ The experiential learning opportunities that our community partners provided were among the major highlights of our curriculum. We worked closely with our community partners to design the experiences so that students would have the best opportunity to see how the SDH affect health. In clerkship evaluations, these activities were often the most highly rated aspects of the curriculum, and we continue to work on adding additional community partners given the positive experiences students reported. The community experiences were also a chance for many students to connect the dots. In required reflection pieces, students discussed seeing the same patients who they cared for in the emergency department or the hospital visiting the soup kitchen where the students worked later that day. As a growing number of health systems are developing strategies to address patients’ unmet social needs, these new partnerships could help identify important areas of community need and guide further instruction for the students. We are also in the process of developing a certificate program that will provide a greater understanding of a broader range of social and economic factors that affect health across all 4 years of medical school.

As we were developing the curriculum, the increase in the pilot curriculum class’s score over time encouraged us to expand the curriculum and add further modules for the next class of students. Interestingly, adding additional modules did not yield higher self-reported knowledge or confidence scores. One possibility is that integrating only some of the modules may be sufficient, which could be an important finding for medical schools struggling to find the time to implement teaching about the SDH.^11,35^ For those wishing to implement the full curriculum, the addition of subsequent modules and experiential activities did not add a significant time burden at our medical school per discussions with students and clerkship directors. A second possibility is that when the full curriculum class received the health equity curriculum, the entire medical school was undergoing a curriculum change that resulted in shorter versions of all third-year clerkships to expand time for clinical electives in the fourth year. It is possible that the students’ reduced clerkship hours affected their knowledge and confidence scores. Third, the survey questions we created may have been too broad to show the outcome of the full curriculum. For example, if we had asked more specific questions about the association of housing instability with health outcomes, it is possible that the full curriculum would have scored higher than the pilot curriculum.

### Limitations

Our study has several limitations. First, as with any single-site study, different schools may see different results. Second, our pre-post design precluded us from determining causality and controlling for any temporal trends in awareness of the SDH. However, because students’ scores increased over the third year and then remained stable for 2 consecutive classes, we believe any significant temporal trends are unlikely. Future studies should evaluate whether changes vary by particular student characteristics (eg, socioeconomic status or geographic areas). Third, we were unable to locate a suitable validated survey and therefore constructed our own. Although we found evidence of internal consistency, our survey has not been validated. Fourth, not every student completed all 3 surveys. There may be some selection bias with students who participated. Additionally, there may be some variability in the teaching some students received as part of their clinical care training that we cannot account for (ie, teaching about health disparities during direct patient care).

## Conclusions

In this cohort study, we found that a dedicated health equity curriculum integrated within the third-year clinical clerkships and partnered with community organizations was associated with an improvement in students’ self-reported knowledge of the SDH and confidence working with underserved populations. The improvement was sustained through the end of school, and students who participated in the curriculum reported higher knowledge and confidence than students who did not. With the growing interest in improving health care professionals’ understanding of the social and economic factors that affect health, integrating a longitudinal health equity curriculum within the third-year clerkships could improve students’ understanding of the SDH and the care provided to underserved populations.

## Notes

Supplement. **eFigure.** Longitudinal Training in Population Health to Reduce Health Disparities **eTable.** Medical Student Knowledge and Confidence With Working With Underserved Populations Survey Items
