# Supplementary material for: Association of a Health Equity Curriculum With Medical Students’ Knowledge of Social Determinants of Health and Confidence in Working With Underserved Populations
Source: JAMA Netw Open. 2021 Mar 1;4(3):e210297. doi: 10.1001/jamanetworkopen.2021.0297 (PMC7921901; doi:10.1001/jamanetworkopen.2021.0297)
Supplement: Supplement. — eFigure. Longitudinal Training in Population Health to Reduce Health Disparities eTable. Medical Student Knowledge and Confidence With Working With Underserved Populations Survey Items [file jamanetwopen-e210297-s001.pdf]

Supplementary Online Content

Denizard-Thompson N, Palakshappa D, Vallevand A, et al. Association of a healthy equity curriculum with medical students' knowledge of social determinants of health and confidence in working with underserved populations. *JAMA Netw Open*. 2021;4(3):e210297. doi:10.1001/jamanetworkopen.2021.0297

eFigure 1. Longitudinal Training in Population Health to Reduce Health Disparities

eTable 1. Medical Student Knowledge and Confidence With Working With Underserved Populations Survey Items

This supplementary material has been provided by the authors to give readers additional information about their work.

© 2021 Denizard-Thompson N et al. *JAMA Network Open.*

eFigure 1. Longitudinal Training in Population Health to Reduce Health Disparities

Wake Forest®
School of Medicine

eFigure 1: **Longitudinal Training in Population Health to Reduce Health Disparities**

Psychiatry Clerkship: Access to Food

Learning Objectives	1. Identify food deserts and their impact on the health of residents of Winston-Salem<br>2. Describe how hunger affects student performance in school
Pre-work	1. Geo-mapping of grocery store options in 2-3 difference addresses
Session	Interactive E-Learning on Food Insecurity<br>- Podcast<br>- Case based format about food insecurity leading to mental health issues, obesity, and other medical problems
Evaluation	1. Turn in Geo Map Assignment

© 2021 Denizard-Thompson N et al. *JAMA Network Open.*

eTable 1. Medical Student Knowledge and Confidence With Working With Underserved Populations Survey Items

1. I am confident about my ability to work effectively with medically underserved
2. I understand the social, economic, historical, political, psychosocial, and cultural factors that influence the health of underserved populations
3. I am familiar with the community resources available to assist members of healthcare teams that treat underserved populations
4. I know how to engage effectively with community and public health teams to reduce health disparities and promote health
5. I feel confident in my abilities to communicate with and assist people with difference needs
6. I understand the role of cross-cultural communication and language skills for community health and education and patient care
7. I feel comfortable negotiating a plan of care with patients from underserved populations, considering their constraints and expectations
8. I have the knowledge and skills required to detect and address most medical problems specific to underserved populations

© 2021 Denizard-Thompson N et al. *JAMA Network Open.*
